# Gastrostomy in the Seventeenth Century

**Published:** 1895-12

**Authors:** F. G. Moehlau

**Affiliations:** Clinical instructor in genito-urinary diseases; instructor in physiology, Medical Department, Buffalo University


### GASTROSTOMY IN THE SEVENTEENTH CENTURY.

By F. G. MOEHLAU, M. D,
Clinical instructor in genito-urinary diseases ; instructor in physiology, Medical Department, Buffalo University.

SOME time ago, an article appeared in several of the papers stating that, in Florence, a man was supposed to have swallowed a table-fork, which statement was greatly doubted. That such accidents are not entirely impossible, the following, given by a member of the University of Königsberg in the seventeenth century, may be of sufficient proof :

History of the Prussian Knife-swallower. By Dr. David Becker :

In the year of our Lord and Saviour Jesus Christ, May 29th, 1635, of the new calendar, a farmer's assistant, A. G., in Gr., situated seven miles from G., felt nauseated in the morning, and, therefore, as he used to force himself to vomit by taking an ordinary knife and, holding it at the tip of the blade, tickling his pharynx with the shaft, he did not have the wished-for result, then, by pushing the knife further down he lost hold of the tip and swallowed it, causing considerable pain before reaching the stomach. He then tried to force back the knife by standing upon his head at intervals. Not being successful in this way, he then drank a gallon of beer and repeated the manoeuvers as before, but with no success. The fear of the knife cutting through the tissues of the stomach must have been intense, yet the poor fellow continued doing his usual work.

After this had been talked about in the surrounding country, the burgomaster heard of it and ordered him to the Rathaus, inquiring into his condition. The burgomaster referred his case to the medical profession of Königsberg, asking for advice in the difficult matter. The reply given was with small encouragement, as such an accident had happened but once before, in Prague ; all historians knew of no other like it.

At Prague, also, a man had swallowed a knife accidentally, and it was taken out again by an artificial cut or opening from the stomach. Therefore, the Königsberg council advised the sending of the sufferer to a conference of the whole medical faculty to decide upon the case, and the result was soon made known by a knighted person to other physicians.

The patient was sent to me, continues the writer, I being dean of the medical faculty at the time. Others also questioned the patient closely in order to understand the whole condition, as I have related before. Upon the advice of the gentlemen, I called a consultation and invited the profession generally. Each one gave his opinion and spoke freely on the subject. Finally we decided that the knife in the stomach must be taken out by an incision, and it seemed necessary to have such work done before the dog-days, and also that the patient should take some balsamic oils, and because the magnetic plaster assisted in the cure of the knife-swallower in Prague it necessarily ought to be tried on our patient. It seemed, finally, that in the cure the Spanish balsam should be employed. To order the surgeon and all necessary for the difficult operation was left to Magister Kruegero.

After the bowels had been emptied by a mild purgative and the balsam given for several days the operation was set for the 9th of July and the work entrusted into the hands of Daniel Schwaben, who is a stone and wound surgeon. As now the external and internal heart tonics, made of pearl water and the like, and other necessary things, were at hand, the beginning of the prayer was made and to God, the Almighty, the most eminent physician and director prayed for success and good progress.

Then the poor victim was tied to a board, the part where the incision should be made marked with charcoal. This was on the left side under the short ribs, two fingers in length. There was skin, the flesh, then the peritoneum, wherein the intestines are situated, to be opened in succession. It was now seen the patient had an empty stomach, that it could not be reached at once, so he was refreshed with pearl water; but the Lord God had mercy and as the stomach was drawn up with a bent needle the surgeon noticed the point of the knife. On this spot the stomach was opened, the knife seized and extracted and wonder it was, the patient exclaimed: "That is my knife!" Soon the patient was released, placed in bed, the wound cleaned and closed with five stitches, but so that some of the balsam could be dropped in; also some peas or turundas immersed in balsam were placed between the stitches. Finally, a cataplasm was applied of bolo, white of eggs and a little alum to keep away the heat. The same day the patient had to be satisfied with a little strengthening soup. At five o'clock he took some of following powders in pearl water mixed with cinnamon water:

R Nutmeg ................................ 2 ozs.
Stone of crawfish (crawfish eyes) ........ $3\frac{1}{8}$ ozs.
Powdered pearls ........................ 16 grs.
S.—Stomach and heart powders.

Thus prescribed for the patient he soon recovered, got married and lived happy.

This report, with a knife, is originally in the library of Königsberg. The table knife measures seven inches in length; the blade is one inch wide.

